# The Tubulin-Based-Polymorphism Method Provides a Simple and Effective Alternative to the Genomic Profiling of Grape

**DOI:** 10.1371/journal.pone.0163335

**Published:** 2016-09-19

**Authors:** Floriana Gavazzi, Luca Braglia, Francesco Mastromauro, Silvia Gianì, Laura Morello, Diego Breviario

**Affiliations:** Institute of Agricultural Biology and Biotechnology - National Research Council, Milan, Italy; Universita degli Studi di Siena, ITALY

## Abstract

The TBP (Tubulin-Based-Polymorphism) method, based on a nuclear ILP (Intron-Length-Polymorphism) molecular marker, has been used for genotyping 37 accessions of the genus *Vitis* inclusive of different species, rootstocks, wild and cultivated subspecies. A distinct DNA barcode made up by a different number of amplicons, was attributed to each of the different accessions. TBP data were compared with those obtained, with the use of an internationally validated set of six SSR markers. Genetic relationships among the different accessions, dendrogram distributions, correlation values and polymorphic index values (PICs) were definitively comparable when not in favor of TBP. Such an experimental consistency is based upon a genomic organization of the multiple members of the β-tubulin gene family, the targets of TBP-mediated amplification, that is conserved in *Vitis* as in any other plant species. The TBP amplicons can actually be used as a useful source of sequence polymorphisms for generating primer pairs capable of identifying specific cultivars in a simple assay. An example for the identification of the ‘Sangiovese’ cv. is reported. More generally, these data are discussed in terms of the actual advantages that the introduction of the TBP method in the field of grape characterization and genotyping can provide.

## Introduction

The grape family (Vitaceae) has an isolated position in the Tree of Life for flowering plants (Angiosperms) but relates to a large monophyletic clade called Rosids that contains other economically important families like legumes (Fabaceae) [1]. The grape family includes five major clades accounting for 900 species divided in 15 genera [2–5]. The genus *Vitis*, accounting for about 70 different species [6, 7], is part of the Ampelocissus-Vitis clade as shown with the use of several and different molecular approaches [4, 5]. According to some reports, the genus *Vitis* can be further separated in two subgenera, subg *Muscadinia* with only two species and subg *Vitis*. This latter comprises all the remaining wild species including rootstocks, and *Vitis vinifera*, the species from which the cultivated grapes *Vitis vinifera* ssp. *sativa* and its closest wild relative *Vitis vinifera* ssp. *silvestris* originated [5, 6]. Both the cultivated species and the wild relatives are considerably variable at genetic level due to their long biological history [8]. Although the biogeographic origin of *Vitis* and its subsequent distribution all over the world remains somewhat controversial [5, 7, 9], reports of *Vitis vinifera* seeds and old winery in the Mediterranean area, with Eastern Turkey as the likely primordial centre of diffusion, consistently dated around the middle of the eighth millennium B.C. [8, 10, 11]. These deep histories are correlated with wealth of broad genetic variability that needs to be tapped by grape geneticists and breeders. Among the grape family, the wild relative species are considered valuable genetic resources of important agronomic traits such as yield, disease resistance and drought tolerance [12]. In fact, the cultivated grapes contain far less genetic variability than that found in the wild species, leaving space for agronomical improvements that may come from the exploitation of potentially useful genes present in the wild species [13].

Ampelography, that is the traditional scientific method based on the description of the morphological characters of the members of the genus *Vitis*, has been used for long time to classify grape species and accessions. It utilizes qualitative parameters to characterize the plant. The efficiency and the importance of this historical method was at last codified by international institutions (i.e. O.I.V Organisation Internationale de la Vigne et du vin/International Organization of vine and wine) allowing to efficiently describe plant morphology of species and cultivars [14, 15]. Ampelometry quantifies some morphological characters of leaves measuring for example length, angle of the nervatures, petiolar senus, width and distance of the leaf lobes [16]. They are important traits useful to describe the accession but not always sufficient to distinguish it, so that many cases of homonymy or synonymy were encountered. In fact, morphological traits can be influenced by the vegetative period, age of the individual, part of the plant, rootstock, and environment. [17, 18]. In addition, ampelography cannot be applied to the identification of juvenile plants. Nevertheless, ampelography is still used on adult plants in combination with the use of molecular markers [19, 20]. In more recent times, as an alternative to the morphological approach, chemical and biochemical methods had increasingly be adopted with remarkable success. Numerous studies have shown that shikimic acid, [21–23], phenolic compounds [24, 25], aroma precursors of berries [26–28], stilbenoids [29] have all a good capacity to characterize the accessions, although not always in a conclusive manner. Interesting results were obtained studying seed proteins. Isoenzymes, additional biochemical descriptors suggested by O.I.V, are also used to a certain extent. Of lately, thanks to the combined use of different ‘omic’ technologies, this biochemical approach has found a renovated consideration [30].

Despite these existing methods, the genetic characterization of grape had the greatest improvement and development in the last two decades. The most important molecular markers applied to the classification of grapevine accessions have been RFLPs (Restriction Fragment Length Polymorphism), RADPs (Random Amplified Polymorphic DNAs), AFLPs (Amplified Fragment Length Polymorphisms), retrotransposon-based markers and microsatellites (SSR, Single Sequence Repeat). By far, SSR markers are now preferred for their high level of polymorphism, reproducibility and codominant features [31]. Since 2004 it was proposed that six internationally accepted informative SSR markers, based on di-nucleotide repeats, should be adopted as a minimal standard marker for the identification of grape cultivars [32]. In order to be effective for identification, allowing inter-laboratory comparison and the establishment of a common database, reference alleles, based on specific cultivars (from here on, cv. or cvs), had to be defined for each of the six loci. Acting as internal size standards, they allow the definition of an homogenous coding system of reference [32]. Subsequent studies, aimed at further increasing the discrimination power of newly conceived sets of SSR markers, were performed. They had either extended the number of the SSRs markers [33, 34] or used markers with a longer core repeat [35, 36]. Also, SSRs have been either used in multiplex reactions or in a sequential order [37] and their discrimination power could be further assisted by the use of retrotransposons [38]. Of lately, although not used as a reference panel for inter-laboratory comparison, a set of 20 SSR markers have been extensively used by several laboratories [39]. These SSRs are mainly based on microsatellite sequences reported in two different studies [40, 41]. In its slightly different versions, the set of 20 SSR markers still comprises 4 of the 6 SSRs (VVMD27, VVMD5, VVMD7, VVS2) of the minimal standard marker mentioned above [32]. Three of them are part of the set of 9 SSR markers that, in a very ponderous and accurate study applied to the characterization of 4370 accessions of the INRA grape repository, was defined as sufficient to distinguish 99.8% of the analyzed accessions [34].

Nuclear DNA-based intron length polymorphism (ILP) markers have never been applied to the genotyping of grapevine and yet, in principle, they could perform successfully because of the high level of heterozygosity, resulting from an elevated outcrossing rate [42]. In addition, since grape does not tolerate long term inbreeding, the grape genome exists in a dynamic state often mediated by transposable elements activity. On the other hand, nuclear introns present in the different members of a gene family are often well dispersed across the genome and quite rich in repeats and transposable elements (TEs), feature that in grape has contributed to the finding of unusual large sizes introns, if compared to plants average [42, 43]. Intron size in *V*. *vinifera* coding DNA sequence (CDS) averages to 213 bp but sizes expansion driven by TE activities, a process that is accelerated by domestication, may lead to intron of several thousand bp in length. Jiang and Goertzen [44] report of uncommonly large size of spliceosomal introns due to the presence of extensive transposable elements, in particular LTR (Long Terminal Repeat) retrotransposon insertions. More significantly, Costa et al. [45] reported that intron2 of VvAox2, a member of the alternative oxidase family, contains a 5028 bp long Ty1/copia-LTR retrotransposon and this feature distinguishes the two clones of the ‘Pinot Noir’ cv. used in the genome sequencing projects [42, 43]. Thus, it was reasonable to investigate about the possibility of using ILP markers for genotyping the genus *Vitis*, at both species and subspecies level, in consideration of some advantages that their use may provide. In fact, ILP markers are neutral, co-dominant, stable, specific since they are tagged to selected genes, successfully applicable at different taxonomical levels and highly transferable among plant species [39]. The assay, based on a simple Exon-Primed Intron-Crossing (EPIC-PCR) reaction that uses one single primer combination for any plant DNA, is fast, convenient, reliable, reproducible, providing a ready-to use, clearly intelligible results [46]. If targeted to the introns of the different members of a gene family, ILP markers work as a very effective multiplex PCR reaction, providing a species or variety specific DNA barcode. One of the most successful ILP marker is that based on the length polymorphism present in the introns of the members of the plant β-tubulin gene family. Named TBP, for Tubulin-Based Polymorphism, has been shown to successfully work on a very large variety of plant species and accessions [47]. In addition to the assignment of a specific barcode, TBP allows the generation of specific molecular probes [48, 49].

The purpose of this work was thus to verify if the TBP method could successfully be adopted for genotyping accessions and cvs of the genus *Vitis*. Application has involved wild species, rootstocks, and either table grape or wine making cvs. TBP data are compared to those obtained, on the same gDNA, with the use of the six SSR sequences of the internationally evaluated set of minimal standard markers. The comparison between the two methods offers several arguments of discussion.

## Materials and Methods

### Plant material

Experiments were performed on 37 accessions of the Riccagioia s.c.p.a. grape vine collection located in the Oltrepò pavese, Torrazza Coste (Pavia), Italy (44°98' N and 9°09' E). The accessions were identified according to the collection name (Table 1) and selected with the following criteria: a group of 4 species of the genus *Vitis* (*V*. *amurensis*, *V*. *arizonica*, *V*. *longij* and *V*. *labrusca* Muncy) with different origin (Asia and America); 2 additional species (*V*. *riparia* NY 245 F15 and *V*. *rupestris*) and their 3 related hybrids (*V*. *berlandieri* Colombard, *V*. *berlandieri* x *riparia* cv. 420A; *V*. *berlandieri* x *riparia* cv. Kober 5bb) commonly employed as rootstocks in grape cultivation; 18 cvs of *V*. *vinifera* ssp *sativa* and their derived clones, including both table grape and wine making accessions. This latter group also includes: an intraspecific cross resulting between ‘Riesling italico’ and ‘Pinot noir’; 3 cvs of *V*. *vinifera* ssp *silvestris* from Tuscany (‘Canalone sotto’), Apulia (‘Sinni 1’), in Italy, and Germany (‘Guemuld 104–64’), respectively. Eight of the 37 analyzed accessions (code 42, K5, BA4, PN1, MR5, CBF, CBS and CHM, in bold in Table 1) were selected for a straight comparison with the SSR assay described by This et al. [32]. Moreover, the following additional cvs and clones were included in the PCR probe assay: ‘Ancellotta’ (A1), ‘Lambrusco Salamino’ (L2), ‘Lambrusco Maestri’ (L3), Sangiovese (SGM) clones AP-SG1, R10, R24, NSG-12T and ULARIS 23.

**Table 1:** List of the 37 analyzed grapevine accessions.

Genus	Species	Sub-Species	Cultivar	Clone	Origin	Aptitude	CODE
*Vitis*	*amurensis*				Asia		AM
	*arizonica*				North America		AR
	*longij*				North America		LO
	*labrusca Muncy*				North America		LB
	*riparia NY 245 F15*				North America	Rts	RI
	*rupestris*				North America	Rts	RU
	*berlandieri x Colombard*				North America	Rts	BE
	*berlandieri x riparia*		**420A**	**MIQ14**	**North America**	**Rts**	**42**
	*berlandieri x riparia*		**Kober 5bb**	**MIK1**	**North America**	**Rts**	**K5**
	*vinifera*	*sativa*	**Barbera**	**R4**	**Piedmont IT**	**Wmk**	**BA4**
				MIB34	Lombardy IT	Wmk	BAM
			Baresana		Apulia IT	Tgr	BRM
			**Cabernet franc**	**VAL—CFV**	**France**	**Wmk**	**CBF**
			**Cabernet sauvignon**	**VAL—CS**	**France**	**Wmk**	**CBS**
				R5	France	Wmk	CBM
			Cardinal		Apulia IT	Tgr	CAM
			**Chardonnay**	**SM113**	**Lombardy IT**	**Wmk**	**CHM**
			Croatina	R2	Lombardy IT	Wmk	CR2
				MI CR 10	Lombardy IT	Wmk	CRM
			Grignolino		Piedmont IT	Wmk	GRM
			**Merlot**	**R5**	**France**	**Wmk**	**MR5**
				R13	France	Wmk	MEM
			Moscato	R2	Piedmont IT	Wmk	MS2
			Nebbiolo	CN 142	Piedmont IT	Wmk	NBC
				R6	Piedmont IT	Wmk	NBR
			**Pinot Noir**	**115**	**France**	**Wmk**	**PN1**
				5V17	France	Wmk	PN5
			Riesling italico	RI-12-V23	Lombardy IT	Wmk	RI2
			R. italico x P. noir	RPN26	Lombardy IT	Wmk	IRP2
			Sangiovese		Tuscany IT	Wmk	SGM
			Syrah		France	Wmk	SIM
			Uva di Troia		Apulia IT	Wmk	UTM
			Uva Italia	Italia VCR5	Apulia IT	Tgr	ITM
				307	Apulia IT	Tgr	REM
		*silvestris*	Canalone sotto		Tuscany IT		SC
			Sinni 1		Apulia IT		SS
			Guemuld 104–64		Germany		SU

Grapevine accessions in common with the study of This et al. [32] are indicated in bold characters. The agronomical aptitude, rootstock (Rts), wine making cultivar (Wmk) and table grapes cultivar (Tgr) is also reported.

### DNA Extraction

For each accession, the genomic DNA was individually extracted from three distinct young leaves of different individual plants and then bulked together as representative of the accession. The DNA isolation method is reported by Lodhi et al. [50] with some modifications hereby described. 0.3–0.5 g of frozen leaves was grinded in liquid nitrogen by addition of 20 mg of PVPP (the highly cross-linked modification of the polyvinylpyrrolidone—PVP) and quartz sand. The powder was resuspended in 5 mL of extraction buffer (25 mM sodium EDTA, 100 mM Tris-HCl pH 8, 2 M NaCl and 3.0% (w/v) CTAB), with a final addition of 10 μL of β-mercaptoethanol. Samples were incubated at 65°C for at least 1h and vortexed every 10 min. Six mL of chloroform:isoamylalcool (24:1) was added and the mixed solution was centrifuged. The top aqueous phase was transferred and the chloroform:isoamylalcool extraction was repeated until the aqueous phase was limpid. After the addition of two volumes of cold isopropanol to the aqueous phase, the sample was stored overnight at -20°C. After centrifugation, the pellet was washed twice with cold 70% EtOH and dissolved in 500 μL of double distilled water. Once re-suspended, DNA was treated with RNAse A following standard operating procedures. DNA quality and amount were determined by both UV absorbance and electrophoresis in 1% agarose gel by comparison with a known amount of Lambda DNA. After electrophoresis, the gel was stained in ethidium bromide solution and photographed under UV light in a gel documentation system (UVIdoc HD2, Uvitec Cambridge UK). Genomic DNA samples were stored at -20°C.

### TBP PCR conditions and amplicon electrophoresis

Fifty ng of genomic DNA was used as a template for TBP 1st intron amplification. The PCR reaction was performed in 30 μl according to Bardini et al. [51]. Primers are those reported in the European patent n.1144691 with slight modifications. The amplicons were preliminarily visualized on a 2% agarose gel. 2 μl of each amplified sample was then loaded on a 6% w/v polyacrylamide native gel and run in 1X TBE buffer (0.089 M Tris-Base, 0.089 M Boric acid and 0.002 M EDTA pH8). Amplicons were visualized by silver staining as described by Bassam et al. [52]. The resulting banding patterns were scanned and the amplicons size were estimated by comparison with a molecular weight marker (Gene Ruler 1kb DNA Ladder Plus—Thermo Scientific) using the Quantity One 1-D Analysis Software (BioRad Laboratories). All PCR reactions were repeated at least twice, to verify the consistency of the TBP amplified products. A negative PCR control (with no template) was also included in each analysis.

Capillary Electrophoresis TBP (CE-TBP) was performed as described in Gavazzi et al. [53] with the following modifications: 2 μl of FAM-labelled PCR product were mixed with 0,18 μl of GeneScan^™^ 1200 LIZ^®^ Size Standard (Thermo Fisher Scientific) and 17,82 μl Hi-Di^™^ Formamide (Thermo Fisher Scientific) to a final volume of 20 μl. After denaturation at 95°C for 5 minutes, the samples were loaded on the Applied Biosystems^®^ 3500 Genetic Analyser (Thermo Fisher Scientific). The CE separation was performed using an 8-capillary array of 50 cm trough the POP-7TM polymer, setting the following instrument specifications: 60°C, injection time 3 seconds, run time 5100 seconds, injection voltage 10 kV and run voltage 8.5 kV. The data collection was performed using the 3500 Series Data Collection Software v. 2.0. (Thermo Fisher Scientific). The data were analysed by the Gene Mapper Software v. 5.0 tools (Thermo Fisher Scientific) and summarized in a Microsoft Office Excel file.

### PCR probes design and amplification

Sequence information was recovered by cloning and sequencing the amplification products obtained by TBP. The isolated target sequences were aligned by BioEdit Sequence Alignment Editor for Window (Version 7.2) and verified according to the sequence information available on NCBI database (NCBI; www.ncbi.nlm.nih.gov/). The primers were manually designed and the reverse oligo was labelled in 5’ position with a FAM fluorophore (Thermo Fisher Scientific). The PCR reaction on genomic DNA, was performed in 20 μl, containing 20 ng DNA template, 1X Taq DNA Polymerase Master Mix providing 2.0 mM MgCl2 (VWR International PBI, Milan, Italy), 0.5 μM of each primers (forward UNITUB8fw TAGCGTATTTGGGAAATTTTRGGTCTG and reverse UNITUB8rev-FAM ATCCGACGCAAAATGCCCAATAC, Thermo Fisher Scientific) and deionized water (Merck KGaA, Darmstadt, Germany). PCR conditions were: 4 minutes initial denaturation at 94°C followed by 35 amplification cycles (94°C for 45 seconds, 62°C for 40 seconds, 72°C for 30 seconds) and final extension step of 30 minute at 72°C. Quantity and quality of the amplified DNA samples were assessed on 2% (w/v) agarose gel. 2 μl containing 100–200 pg of amplified labeled DNA were mixed with 0.22 ul of GeneScan^™^ 500 LIZ^™^ Size Standard (Thermo Fisher Scientific) and 17.78 μl Hi-Di Formamide to a final volume of 20 μl. After denaturation at 95°C for 5 minutes, the samples were loaded on the Applied Biosystems^®^ 3500 Genetic Analyser. CE separation was performed using an 8-capillary array of 50 cm trough the POP-7TM polymer, setting the following instrument specifications: 60°C, injection time 5 seconds, run time 1680 seconds, injection voltage 10 kV and run voltage 15 kV.

### SSR

25 ng of genomic DNA was used as a template for PCR amplification. Six microsatellite markers were chosen as a core set according to the international grape genetics community [32]. The PCR primer combinations referring to the SSR loci VVMD5, VVMD7 [40], VVMD27 [54], VVS2 [31], VrZAG62 and VrZAG79 [55] were used and the reaction conditions were carried out according to the PCR protocol and conditions described by This et al. [32]. The PCR amplified products were preliminary checked on a 2% (w/v) agarose gel. 2 μl of each PCR product was then loaded on a denaturing polyacrylamide gel (6% polyacrylamide, 7 M urea). Before loading, each sample was denatured for 3 min at 94°C with the addition of an equal volume of formamide buffer (100 ul 1M NaOH, 400 ul H2O, 9.5 ml formamide, 50 mg Bromophenol Blue, 50 mg xylene cyanol). A 50 bp DNA ladder (GeneRuler^™^ 50bp DNA Ladder—Thermo Fisher Scientific) was used to estimate amplicons sizes. Electrophoresis was performed in 1X TBE buffer at 1500 V for 3 hours. At the end, the amplified fragments were visualized by a silver-staining procedure, according to Bassam et al. [52] protocol. The amplicons size and score were estimated by using the Quantity One 1-D Analisys Software (BioRad Laboratories). All the PCR reactions were repeated twice at the least, to ensure consistency of the SSR-PCR amplified products. Negative (no template) and positive controls (25ng of *Glycine max* gDNA) were also included in the analysis.

### Data Analysis

The amplified fragments generated by both the TBP and the SSR markers were scored and used to produce a presence/absence matrix. The genetic similarity values were estimated, among the analysed genotypes, using the NTSYSpc 2.1 software, according to the Nei and Li coefficient [56, 57]. Dendrograms were computed using the TREE procedure of the NTSYS 2.1 software applying the unweighted pair group method with arithmetic mean (UPGMA). The Mantel test [58] obtained using the COPH and MXCOMP procedures, was used to verify the level of agreement of the UPGMA dendrograms to the respective TBP or SSR similarity matrix. The statistical confidence of a particular group of accessions in the tree was evaluated by bootstrap test [59] determined by the TREECON program for Windows [60]. The similarity matrix was also subjected to Principal component analysis (PCA). The Polymorphism Information Content (PIC) values were calculated using the formula of Anderson et al. [61].

## Results

### TBP and SSR analysis

**Fig 1:**
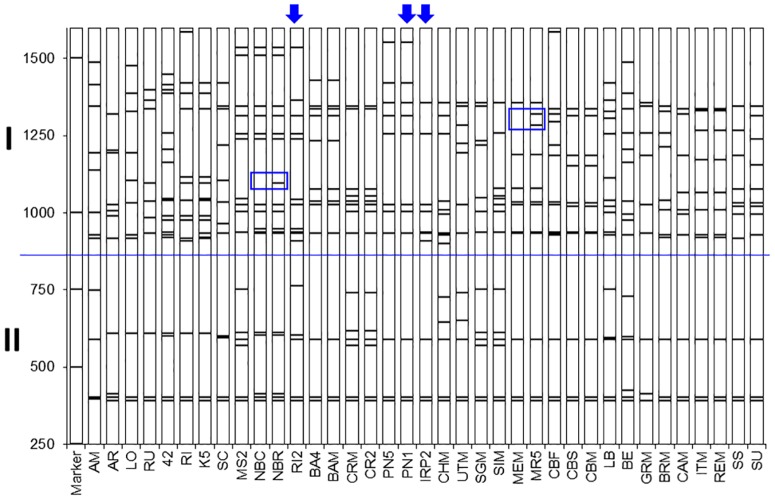
TBP genomic profiles. TBP amplification pattern of the 37 grapevine accessions of Table 1. Cvs are ordered according to cluster analysis of Fig 2. Blue boxes indicate few polymorphisms associated to clones of the same cv. (NBC vs NBR and MEM vs MR5). Arrows on top indicate the profile of the IRP2 cross and its parentage (PN1 x RI2); I and II indicate TBP-amplified products containing introns longer or shorter than 500 bp; Molecular sizes marker is reported on the left.

**Fig 2:**
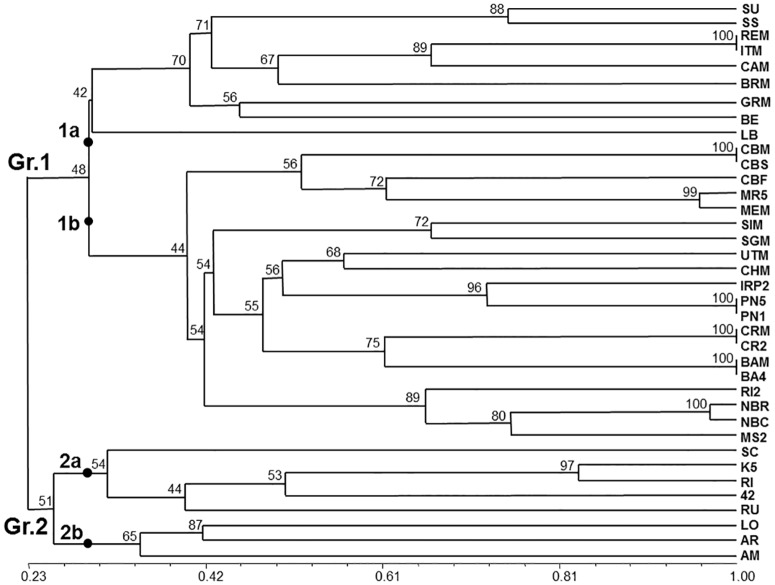
TBP derived cluster analysis. Unweighted pair group method with arithmetic mean (UPGMA) tree based on Nei and Li’ genetic distance calculated on the TBP data of 37 grapevine accessions. The bar at the bottom of the figure provides a scale, from 0 to 1, of the estimated similarity. The numbers next to each node represent the bootstrap value estimated for 1000 replicates.

Fig 1 shows the polymorphic TBP profile of the β-tubulin 1^st^ intron obtained through the amplification of genomic DNA extracted from the 37 different grapevine accessions of Table 1. Profiles are reported in the same order as that resulting from the cluster analysis of Fig 2. Each analyzed accession shows its own specific, exclusive amplification pattern with the exception of the clones that originate from the same cv. (‘Barbera’, ‘Croatina’, ‘Nebbiolo’, ‘Pinot Noir’, ‘Merlot’, ‘Carbernet sauvignon’ and ‘Uva Italia’) that share an almost identical banding pattern. Exceptions, limited to 1 or 2 additional bands, are observed in the ‘Nebbiolo’ and ‘Merlot’ samples, respectively (blue boxes in Fig 1). Each amplification profile is constituted by a different number of fragments, with size that goes up to 1500 bp. Their length reflects the contribution of both the amplified introns and the 305 bp long exon flanking region. In accordance, deduced intron sizes can fluctuate from few tens to more than 1200 bp. As shown, the first intron of the *Vitis* β-tubulin genes distributes quite sharply in two major groups (I and II in Fig 1) characterized by intron sequences longer or shorter than 500 bp, respectively. By far, group I shows the highest level of polymorphism. A wide range of allelism can be appreciated with a number of amplicons that can be as low as 10, chiefly the group of the table grapes or as high as 18. Ten is the number of the β-tubulin genes reported by the genome sequencing project [43]. These findings are consistent with molecular data we have initiated to collect from few cvs by the molecular cloning of their TBP-amplified products (see below and discussion). In addition, the tubulin identity of the amplification products was assessed by comparing the nucleotide sequence of the exon fragments that were amplified by TBP with the corresponding sequence of a rice tubulin isotype (OsTub4; ID X78143) arbitrarily chosen as an external control (S2 Fig). Nucleotide homology is no less than 75% while the corresponding homology at amino acid level is almost 100% (S3 Fig).

A binary matrix resulting from the score for presence/absence of the TBP amplified products was used to obtain the dendrogram of Fig 2. As shown, the TBP marker revealed the presence of a broad range of genetic diversity, with an estimated similarity value that ranges from 0.26 to 1. The highest values are estimated between clones of the same cv. that show an almost identical amplification pattern with the exception of the two ‘Merlot’ and the two ‘Nebbiolo’ clones (blue box in Fig 1) that can be further recognized for the presence of 2 or 1 differentiating bands, respectively. The phylogenetic tree distributes the analyzed samples in two major clusters separating the *V*. *vinifera* accessions (Group 1) from all the other species (Group 2) with few exceptions (see below and discussion). In Gr. 1, two subgroups, 1a and 1b, can clearly be distinguished. Subgroup 1a is the most heterogeneous. It contains all the table grape cvs (‘Uva Italia’, ‘Cardinal’ and ‘Baresana’) that, although morphologically different, share a similar TBP profile, the isolated ‘Grignolino’ (GRM) accession and the roostock *V*. *berlandieri* x Colombard (BE) resulting from the interspecific hybridization of *V*. *berlandieri* and *V*. *vinifera*. Two subspecies of *V*. *vinifera* ssp. *silvestris* (SU and SS) also cluster within this subgroup.

Subgroup 1b contains different clusters that separate, in a quite orderly way, all the remaining wine making cvs: one group of French origin (‘Cabernet’ and ‘Merlot’); a couple of cvs from Lombardy (‘Barbera’ and ‘Croatina’); one group from Piedmont (‘Nebbiolo’ and ‘Moscato’), the ‘Pinot Noir’ samples (PN5 and PN1) and the two cvs originated by them: IRP2 (‘Riesling italico’ x ‘Pinot Noir’) and Chardonnay (‘Pinot noir’ x ‘Gouais blanc’). With reference to Gr 1, the correlation between the data of the binary matrix and dendrogram elaboration is highly significant with r = 0.86742. Concerning Gr.2, that includes only species distinct from *V*. *vinifera* ssp. *sativa*, the two species of North American origin (LO and AR) cluster together with *V*. *amurensis* (AM), from Asia, as the most isolated subgroup of the tree. In a separated subgroup the rootstocks species (RI and RU) give rise to a recognizable cluster together with the derived hybrids (42 and K5). On the contrary of the two other *V*. *vinifera* ssp. *silvestris* (SU and SS), SC segregates in Gr. 2 although in a rather isolated branch.

A correlation value r = 0.86416, obtained from comparing the raw data of the matrix and the derived dendrogram, was calculated for all the 37 species analyzed with TBP. This value is highly significant and higher than the corresponding value of r = 0,82118 we obtained by analyzing the same samples with the standard set of the six internationally recognized SSR markers (S1 Fig).

The two dendrograms obtained either by using TBP or the set of SSR markers, show confirmations and differences. These latter are mainly restricted to the group of table grapes that appear more spread in the SSR-based dendrogram.

As already anticipated, our sample size included 8 accessions that were previously characterized by the use of the internationally recognized, standard set of 6 selected SSRs markers [32]. With the aim of comparing the genetic distribution obtainable from the two methods on the same material, the same SSR set was also applied in our laboratory. The estimated sizes of the amplified SSR alleles were fully consistent with those reported by the 10 laboratories of the international consortium (S1 Table). In fact, the sizes of each allele fall within the defined, acceptable interval of fluctuation. This can be further appreciated in Table 2 where the frequency and the sizes of each specific allele are reported for the whole set of the six SSR markers.

**Table 2:** Size and relative allele frequency values of six SSR polymorphic loci.

VVMD05		VVMD07		VVMD27		VVS02		VrZAG62		VrZAG79	
Size	f	Size	f	Size	f	Size	f	Size	f	Size	f
226	0,2500	231	0,0625	170	0,0625	129	0,0625	188	0,1875	239	0,0625
228	0,0625	233	0,0625	182	0,1250	135	0,0625	192	0,1250	243	0,1250
232	0,0625	239	0,3750	186	0,1250	137	0,0625	194	0,3125	245	0,1250
234	0,0625	243	0,1250	190	0,3750	139	0,1875	196	0,1250	247	0,1875
236	0,1250	247	0,0625	192	0,1250	141	0,1875	200	0,1250	251	0,0625
238	0,1875	249	0,0625	194	0,0625	143	0,0625	204	0,0625	255	0,1250
240	0,1250	253	0,0625	212	0,0625	145	0,0625	216	0,0625	259	0,3125
264	0,0625	263	0,1250	218	0,0625	149	0,0625				
266	0,0625	265	0,0625			151	0,0625				
						153	0,1875				

SSR allele size (base pairs) and estimated frequency (f) used by the International consortium [32] to identify eight grapevine cvs common to this study.

**Fig 3:**
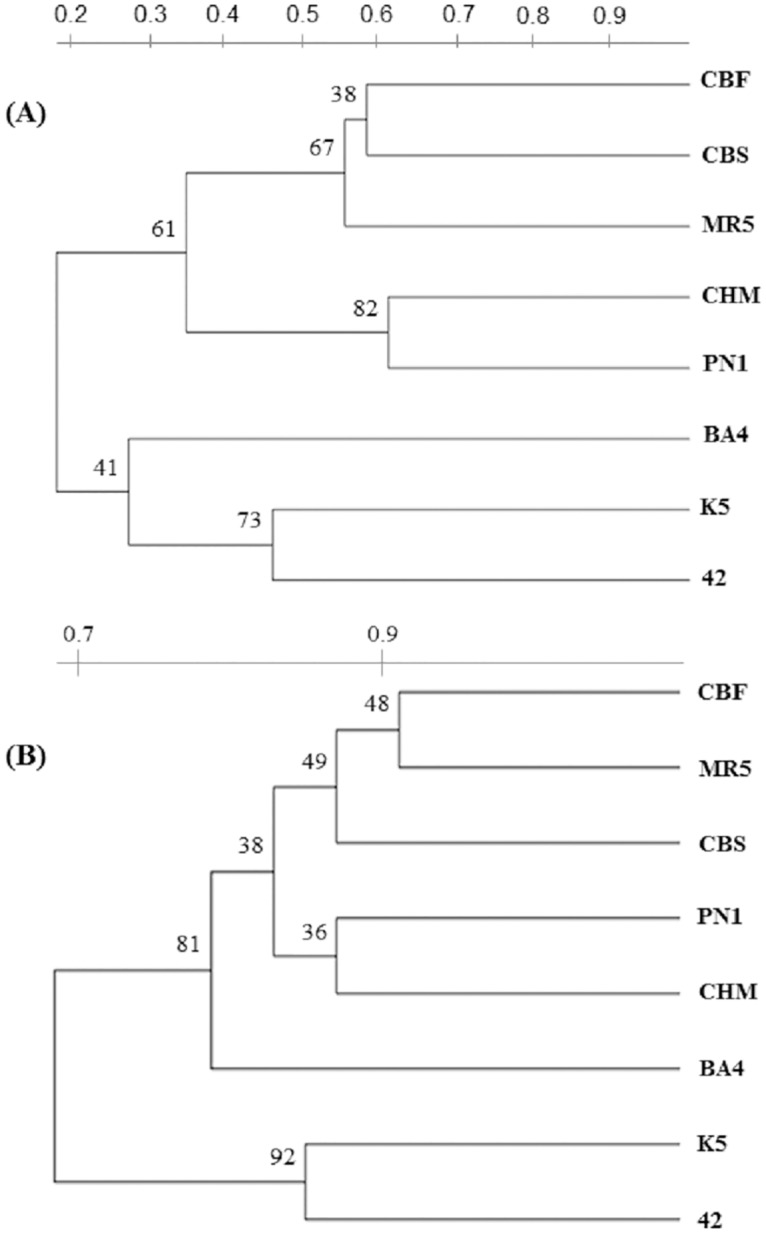
TBP versus SSRs comparative cluster analysis. Dendrograms of eight selected cvs produced from either SSRs (A) or TBP (B) data. Grapevine cvs are those in common with the International consortium study [32]. The cv. code refers to the accession name reported in Table 1. The estimated similarity value, ranging from 0 to 1, is reported on bars at the top of each dendrogram.

Comparison between the two methods, TBP or the SSRs set, was further assessed by analyzing the distribution of their specific dendrograms. As shown in Fig 3, the similarity of the distribution was just broken by the positioning of the Italian BA4 cv. that, in the TBP derived dendrogram groups together with the *V*. *vinifera* cvs whereas, when using the SSRs set, is more closely associated to the two rootstocks (K5 and 42) clearly separated from the main cluster, in both dendrograms. On the same line of comparison, we have also evaluated the level of polymorphism present among the 37 grape samples by calculating the PIC values for both the TBP and the SSR loci (Table 3). An overall number of 113 rather than 117 alleles were detected at the TBP or the SSR loci respectively. The PIC value, that provides a measure of polymorphism for a marker system by reflecting the degree of allele diversity and its frequency among accessions, was also independently estimated for species and subspecies (Table 3). The PIC data indicate that the TBP technique has a comparable discrimination power as the six SSRs, since the PIC value for TBP should actually be referred to the total number of alleles, lower than that of the SSR set. In both marker systems, a lower PIC value was found for the group of the *V*. *vinifera* ssp. *sativa* subspecies that includes the higher number of clones, often indistinguishable from each other for their molecular pattern.

**Table 3:** Comparison of the estimated PIC values calculated for the two different marker systems, TBP and SSR.

			PIC			
		allele n°	37	VSPP	VV	VVSa
**TBP**		113	0,974	0,973	0,973	0,971
	***VVMD5***	19	0,916	0,895	0,913	0,899
	***VVMD7***	20	0,920	0,932	0,901	0,898
	***VVMD27***	20	0,914	0,914	0,903	0,894
**SSR loci**	***VVS2***	21	0,913	0,929	0,894	0,891
	***VrZAG62***	19	0,921	0,901	0,889	0,886
	***VrZAG79***	18	0,928	0,928	0,924	0,918
	***mean***	20	0,919	0,917	0,904	0,898
	**tot.**	117	0,986	0,986	0,985	0,983

PIC (Polymorphism Information Content) values estimated among: all the analyzed grapevine accessions (37); species different from *V*. *vinifera* (VSPP); all the accessions of *V*.*vinifera* (VV) and just the *V*.*vinifera* subspecies *sativa* (VVSa). The PIC value was calculated from both mean and total number of the 6 SSR loci.

### Comparison between CE-TBP profile and annotated genomic sequences of ‘Pinot Noir’

**Fig 4:**
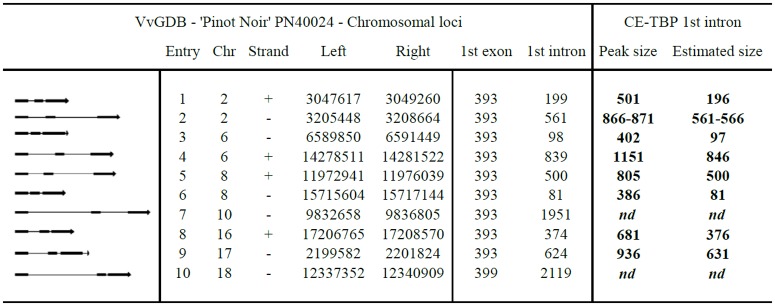
Genomics of the *Vitis vinifera* β-tubulin loci with reference to the first intron sizes. *Vitis vinifera* β-tubulin genes first intron size: comparison between nucleotide sequence information ('Pinot Noir' PN 40024) retrieved from *V*. *vinifera* Genome Data Base (Genoscope 12X—Assembly version 12 Feb 2010) and experimental data obtained with CE-TBP. The first column on the left reports the schematic representation of the genomic organization of the β-tubulin genes in *Vitis*. Introns, represented by lines, are interspersed within exons, represented by black boxes. The last columns on the right report the size of the amplified CE-TBP fragments and deduced 1st introns length, obtained from cv. PN5 ('Pinot Noir' 5V17). Chromosome number (Chr), strand, nucleotide number (left and right border) and the length in base pairs of the 1st exon and 1st intron are reported for each entry. Peak size, in base pair for each β-tubulin gene, is detecdet by the CE-TBP 1st intron method; estimated size of the 1st intron is calculated by subtracting 305 bp from the Peak size; nd not determined.

Reliability of the information based on the application of the TBP marker has been also assessed in comparison with the genomic data released by the whole genome shotgun project that used ‘Pinot Noir’ (PN40024) as the cv. of reference because of its high level of homozygosis. Nucleotide sequence information, retrieved from the *V*. *vinifera* Genome Data Base (VvGDB, version Genoscope 12x) through the plant Genome database browser (http://www.plantgdb.org/VvGDB) and relevant to this study is reported in Fig 4 together with a graphic representation of the ten annotated β-tubulin genomic loci. This figure shows that, similarly to other plant species, the genomic organization of the β-tubulin genes is characterized by the presence of two introns of different length located in conservative positions of the exon coding sequences retrieved.

The capillary electrophoresis TBP variant (CE-TBP) was used as the method of comparison. Performed under denaturing condition, CE-TBP provides more accurate molecular data then PAGE separation with reference to the actual sizes of the amplified fragments [53]. However, we could not use CE-TBP for marker analysis because this technique, in our hands, cannot provide a reliable resolution for fragments higher than 1200 bp in size, limit over which numerous alleles can still be detected. Nevertheless, comparison of the data obtained with CE-TBP on ‘Pinot Noir’ (PN5) with respect to 8 of the 10 genomic entries provides some additional, interesting information (Fig 4, right part). First, the CE-TBP technique looks remarkably precise with reference to the number and size of β-tubulin introns, this latter deduced after subtracting 305 bp of the amplified flanking exon sequences. In fact, intron sizes, although obtained with a much more simplified procedure, very closely match those retrieved from the sequencing project database. In addition, a clear biallelism at entry 2, not reported in the VvGDB, could be consistently identified. It refers to two peaks with sizes of 866 and 871 bp respectively. They correspond to introns of 561 and 566 bp, both likely related to the intron of 561bp of Entry 2 (Fig 4). This finding, together with the numerous and different alleles uncovered in the other cvs, just indicate that the search and characterization of DNA polymorphism in grape remains an important issue, more so for a sequence tagged marker as it is TBP.

### Development of a cultivar specific probe

**Fig 5:**
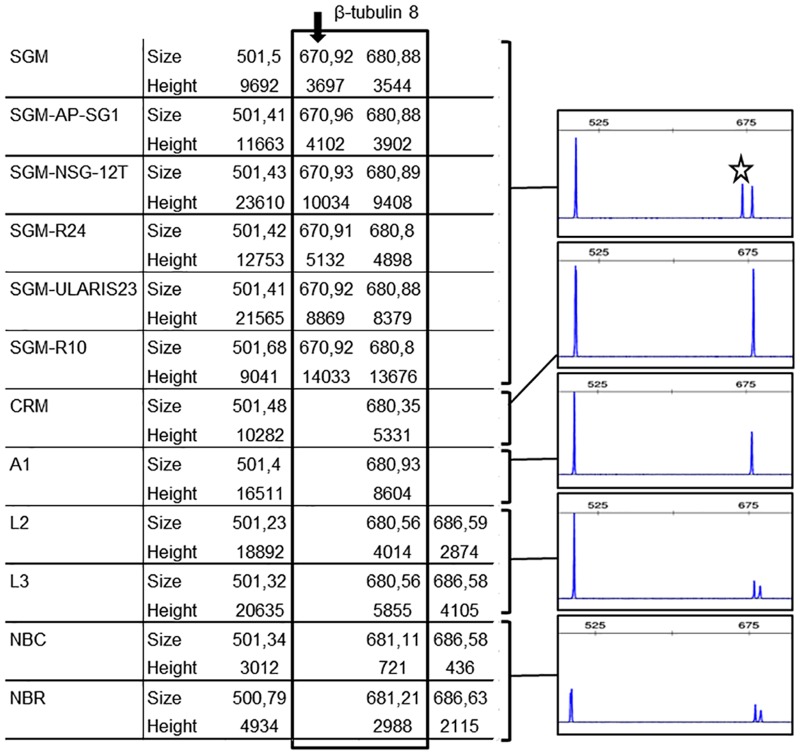
A doublet characterizes the ‘Sangiovese’ (SGM) cv. On the left: CE-TBP values (peak size in base pair and peak height in Relative Florescence Unit—RFU) obtained from the amplification of the first β-tubulin intron, relative to entry 8 of the *Vitis vinifera* Genome Data Base (VvGDB, version Genoscope 12x). The box highlights the 670–680 bp specific doublet of the SGM cv., detected in all analysed clones. The SGM specific peak of about 670 bp is further highlighted by the arrow. On the right, the corresponding portion of the electropherogram are reported for each analysed cvs (‘Sangiovese’ (SGM); ‘Croatina’ (CRM); ‘Ancellotta’ (A1); ‘Lambrusco’ (L1, L3) and ‘Nebbiolo’ (NBC, NBR)). The star points to the specific 670 bp peak of ‘Sangiovese’ clones.

**Fig 6:**
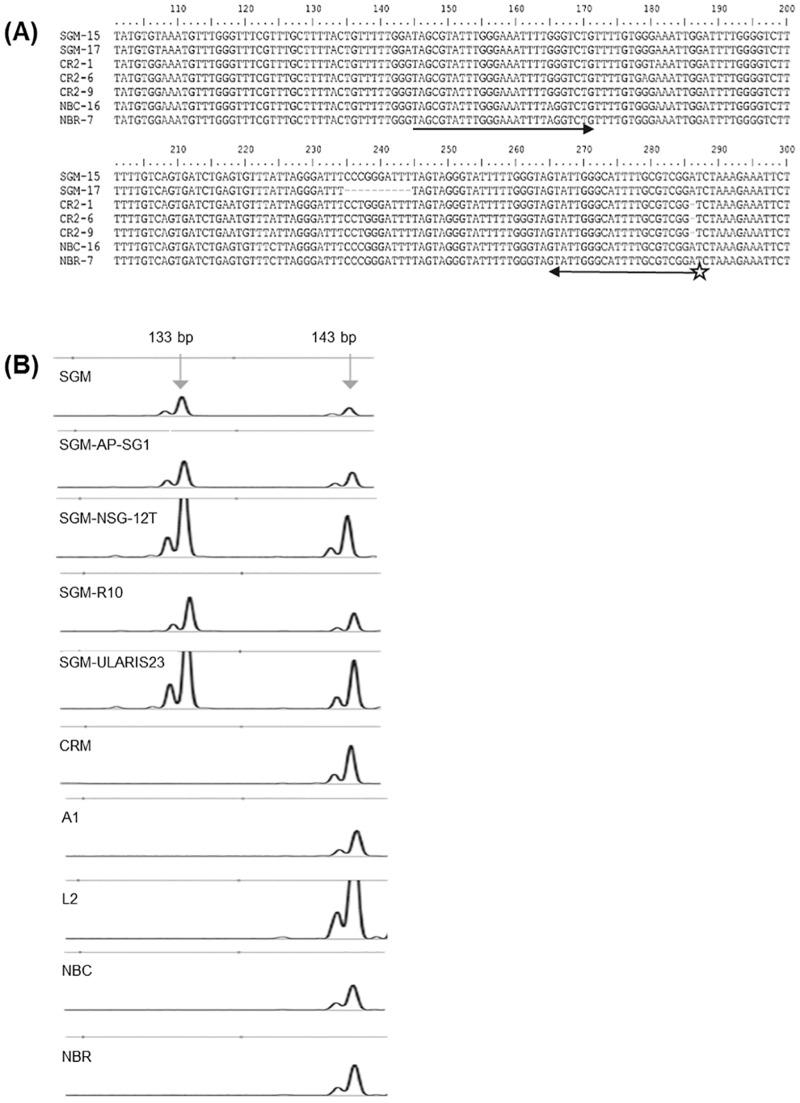
Molecular basis of the 'Sangiovese’ (SGM) cv. specific polymorphism. (**A**) Sequence alignment of portions of the β-tubulin genes of three wine making cvs (‘Sangiovese’, ‘Croatina’, ‘Nebbiolo’), with the corresponding entry 8 of the *Vitis vinifera* Genome Data Base (VvGDB, version Genoscope 12x). Different molecular clones of the same cv. are indicated by different numbers. A 10 bp deletion is found in the SGM-17 clone. The arrows underscore the sequences used to design the forward and reverse primers and the star represents the fluorophore marker linked at 5’ position. (**B**) Portion of the electropherogram obtained by resolving, through capillary electrophoresis, the size of genomic DNA fragments amplified with the primer pair reported in (**A)**. A specific doublet of 133–143 bp is detected only in the ‘Sangiovese’ (SGM) clones.

TBP offers the advantageous possibility of developing highly specific molecular probes that can be used for cultivar recognition and wine and must authentication. The high level of allelic polymorphism present in each grapevine accession provides the basis for this strategy. As a proof of principle, we have developed a simple end point PCR approach for the easy recognition of the ‘Sangiovese’ cv. (SGM), the most largely cultivated Italian wine making grape from which prestigious brands such as Brunello di Montalcino are obtained. In order to develop a simple assay we concentrated our interest on the allelic polymorphism uncovered by the CE-TBP method around 680 bp sizes, the peak corresponding to entry 8 in PN40024 (Fig 4). In addition to this peak and differently from all the other wine making cvs analyzed, ‘Sangiovese’ contains an allele that is 10 bp shorter (Fig 5), as also confirmed by sequencing of the corresponding fragment (Fig 6A). The 670 and 680 bp doublet looks specific for ‘Sangiovese’ since it is found in several different clones (Fig 5). Molecular cloning and sequence comparison between the introns corresponding to β-tubulin entry 8 of different wine making cvs allowed the design of a pair of primers, one of which labeled with a fluorophore (Fig 6A), that could easily and conveniently amplify the doublet specific for SGM, in a simple PCR assay followed by capillary electrophoresis analysis (Fig 6B). Ultimately, the presence of the 10 bp deletion in the SGM was confirmed by the nucleotide output of a massive sequence project done on all the TBP fragments generated by this specific cv. (data not shown).

## Discussion

SSRs undoubtedly represent a very useful molecular tool that, either alone or in combination with additional approaches such as ampelography, use of retrotransposons or nucleotide sequencing, can assess genetic diversity in cultivated grape, wild species and rootstocks, solve homonymy and synonymy issues, identify hybrids parentage, defining the internal genetic relationships among genotypes of large germplasm collections. The robustness of the systems of identification based on SSRs has been progressively implemented by augmenting the number of SSRs that are concomitantly used rather than increasing the length of their core, repeated unit. Of lately, there seems to be a general agreement on the use of a set of 20 SSRs for providing the most reliable information [39]. Laucou et al. [34] further limited this group to 9 SSR markers effectively capable of distinguishing 99.8% of the grape accessions. The 20 SSRs markers set originates from the combination of microsatellites used in two previous pivotal studies performed by Adam-Blondom et al. [62] and This et al. [32]. This latter is of particular relevance since a set of 6 SSRs based on dinucleotide repeats was successfully shared as a reference panel by an International consortium of laboratories and this remains up to now the only interlaboratory validation accomplished in *Vitis* genotyping. 4 of these SSRs (VVMD5; VVMD7; VVMD27 and VVS2) are still present in the group of 20 and 3 of them in the subgroup of 9.

Since we were aiming at verifying the level of reliability of TBP with respect to an SSR-based approach, we used the same 6 SSRs reference panel for genotyping those cvs that were in common between our and the international consortium study. As a matter of fact we acted as an additional external laboratory producing SSR data perfectly consistent with those reported by the International consortium thus providing a correct ground for comparison. Although very powerful, as reported, microsatellites remain anonymous markers subjected to a high degree of genetic hyper-mutability that, based on impaired DNA mismatches repair, is likely independent from the stabilization of morphological features and traits of agronomical relevance. It is under this view that some difference observed by comparing the dendrograms derived either from the TBP or the SSR markers can likely be explained. In addition, due to some variability in size determination that may occur in different laboratories, depending on different reagents, instruments and PCR conditions, reference alleles for each microsatellite must be designated to strengthen inter-laboratory data comparability. Moreover, the amount of data generated by the use of SSRs in large size samples is relevant and requires adequate software and computer sciences facilities to be processed, conditions not always available in research laboratories. Given all this, we investigated if a completely different molecular marker based upon Intron Length Polymorphism (ILP) could represent a valid option to grape genotyping and classification by providing reliable information, comparable to that obtained with the SSRs, with a reduced operation time and no need for handling a massive amount of data.

TBP is a quite successful ILP marker capable of identifying DNA length polymorphisms that occur in the two introns present within the coding sequences of the different genes that constitute any plant β-tubulin gene family. This is possible because the position of the two introns is strongly conserved among all the different plant species and the exon boundary sequences exhibit a limited amount of variation allowing the design, and use in PCR reactions, of degenerated primer pairs that are plant specific. This same genomic organization is also found in *V*. *vinifera*, as inferred by the whole genome sequence, stored in the VvGDB (Fig 5). Ten different members of the β-tubulin gene family have been identified. All of them contain two introns of variable sizes located at the conservative positions 393 and 662 of the coding sequence with the exception of entry 10 that contains two additional codons in the nucleotide region corresponding to the protein N-ter, irrelevant for the analysis. TBP is also a codominant marker thus allowing the detection of both alleles of a single β-tubulin locus. This is a feature of relevance, in presence of a high level of heterozygosity, as it is the case for grape, easily appreciable in any of the data we have presented where a vast fluctuation in the number of the products amplified from the 37 different accessions is shown. This also reflects a high level of DNA polymorphism, a considerable multiallelism that does not find correspondence in the molecular data retrievable from the data base of the grapevine genome sequencing project. Significantly, an undocumented biallelism occurring at the level of entry 2 was even found in ‘Pinot Noir’ (PN5), a clone of the same highly homozygous cv. chosen for the whole genome sequencing project. In fact, two peaks corresponding to introns of 561 and 566 bp respectively were found in place of the single one of 561 bp found in the VvGDB (Fig 5). Such minimal discordance in sizes is well over the limit of CE-TBP resolution that is +/- 1.5 bp. TBP, based on target sequences largely shared within the plant kingdom, most typically attributes a specific multiple bands code to any plant species, with no need for any preliminary information on the genome to be analyzed. In other words TBP always photographs a distinguished DNA polymorphisms at species level. This may not always occur at variety levels: it depends from the biological origin, the history of species propagation and the frequency of cross pollination between different varieties. Typically, highly allogamous species are easily fingerprinted by TBP. Domesticated grape is highly cleistogamous and has been mainly agamically propagated for more than 8000 years. This means that cross pollination, either natural or supported by man, has contributed very little to the existing genetic variability. Yet, what TBP has highlighted is an extensive polymorphic pattern occurring at cultivar level. In fact, each cv. shows its own specific TBP profile while a substantial identity in the banding pattern has been observed only among clones. Cipriani et al. [36] also stated that parentages and kinships not known from historical records can only be discovered through massive genotyping of large collections and this would be particularly true for wine grape varieties with an unknown origin, grown for centuries. If applied in this context, TBP may save a lot of time and, even when not decisive, can certainly provide a rather accurate first ordering of the data in groups that share identical or closely related pattern. In fact, we have recently been able to genotype a small ligneous specimen of a shoot found in an Etruscan necropolis to find out that its TBP profile overlapped with that of the ‘Sangiovese’, a cv. typically grown in that area of Tuscany, since long time (data not shown). The thousands of cvs not previously identified may also quickly benefit from a systematic application of the TBP-based genomic profiling.

TBP data can be analyzed and discussed with reference to three different levels of comparison. First, in relation to the SSR data obtained on the 37 different grapevine accession. Second, in relation to the data obtained by the International Consortium of laboratories on the 8 accessions common to both studies. Third, with reference to the available genome sequencing data. The cluster analysis performed with the TBP data, further supported by PCA (data not shown), grouped the 37 analyzed samples in a way that is comparable to SSR's if not more consistent with respect to some taxonomic and agronomic trait. In fact, the TBP method clusters together the majority of the grapevine species and rootstocks (group 2) while they are scattered across the tree when using the SSRs set. Table grapes group together with TBP but separately when the SSRs are used and this occurs even with the 34 SSRs set [36]. Both methods cluster together cv. of French source (CBM, CBS, CBF, MEM and MR5) highlighting a strong, common origin, and place the IRP2 cross close to its PN5 parent. This is likely due to the high level of allelic polymorphism found by TBP in RI2, characterized by 16 different bands (Fig 1). Likewise, when comparing the 8 accessions used in the International laboratory comparison [32], the two trees look very similar (Fig 4), despite the overall small size of the sample. In fact, the TBP analysis clusters the BA4 cv. together with the other *V*. *vinifera* accessions whereas the same cv. remains separated in the tree derived from SSRs. Given these differences and in consideration of the calculated PIC values, the conclusion can be drawn that the information provided by TBP is at least as reliable as that obtained with the use of the international recognized standard set of SSR markers. At practical level, several advantages related to the use of TBP marker should instead be considered. TBP save time and reagents, since the same pairs of primers is used in a single PCR reaction, no matter what genome neither which taxonomical level is under investigation. When using the 6 SSRs standard set we had to run 6 independent PCR reactions. Now several studies using a higher number of SSRs have found experimental conditions that allow to perform multiplex PCR reactions but still they remain a few, requiring multiple sequencer runs. In addition, TBP does not require any reference allele since samples are recognized and discriminated for their profile made up by multiple bands. Because of its simplicity TBP doesn't require any sophisticated computing elaboration but a simple data base, storing the profile of all the analyzed accessions, can be constructed and used to verify the identity of new samples. This would also simplify the genotyping of large collection of species and varieties belonging to the *Vitis* genus, largely reducing the systematic repetition of the analysis and defining more relaxed relationships such as kin groups, co-ancestry and inbreeding. When used for parental identification, TBP can clearly verify if the hybrid originate from the contribution of the two putative parental lines, as shown here for the IRP2 and previously for some *Jatropha curcas* accessions and *Passiflora* spp.[63, 64]. TBP can also contribute to the rapid and easy recognition of juvenile plants, impossible to be identified in the first 3 or 4 years of growth because of the lack of those morphological traits that typically characterize adult plants. This has also important consequence on the timely prediction of the identity, quality and quantity of grape production.

Finally, an additional advantage that can be attributed to TBP is the design and production of primer pairs that are specific for any cv. or accessions of interest. In fact each TBP-amplified band may potentially contain a distinguished sequence information. Working on the amplified bands that, in the different wine-making cvs of this study, correspond to the β-tubulin entry 8 of the VviGDB, we have designed and used a couple of primers capable, in a simple PCR reaction, to amplify a doublet that is specific for ‘Sangiovese’ (SGM), the grapevine cv. more widely cultivated in Italy, used for making the most appreciated brands of wine. This is just an example that can be followed up by the preparation of additional and numerous variety specific assays, useful for cv. recognition rather than must characterization. Referring to this latter, and wine in perspective, the possibility of amplifying a short size diagnostic fragment, as shown for SGM, is of importance in consideration of the difficulty that is encountered in DNA isolation, typically degraded and present in low abundance. In conclusion, this paper provides a series of data and evidence in favor of the argument that TBP is a useful, rapid, simple, reliable molecular tool that can be used, either alone or in combination with other markers, for different purposes that may include establishing the genetic relationships within the grape family Vitaceae, barcoding wild and cultivated *Vitis* accessions including rootstocks, genotyping grapevine collections and developing variety-specific assays.

## Supporting Information

S1 Fig SSRs derived cluster analysis. Dendrogram of the 37 grapevine accessions based on SSR marker analysis. The bar at the bottom of the figure provides a scale, from 0 to 1, of the estimated similarity. The numbers next to each node represent the bootstrap value (1000 replicates).
(TIF)

S2 Fig Nucleotide sequence comparison of the tubulin exon portions amplified by TBP. Exon nucleotide sequences amplified by TBP are aligned using rice OsTub4 as reference. The sequence corresponding to the primer pair (Fex1 and Rex1) is shown at the two opposite ends while an arrows indicate the position of intron1.
(TIF)

S3 Fig Deduced aminoacid sequence comparison. The tubulin exon portions shown in S2 Fig have been translated into the corresponding aminoacid sequences.
(TIF)

S1 Table List of the reference alleles of the six internationally validated SSR loci. Comparison of the six SSR alleles size obtained in this study (indicated by * in bold character) with those reported by the ten laboratories (lab. 1–10) of the International consortium [32], on the same grapevine cvs. The size of both alleles (a and b), expressed in bp, is reported for each of the six different SSR loci. Not determined allele size is indicated by nd.
(XLSX)
