# Supplementary figures and images for: The Tubulin-Based-Polymorphism Method Provides a Simple and Effective Alternative to the Genomic Profiling of Grape
Source: PLoS One. 2016 Sep 19;11(9):e0163335. doi: 10.1371/journal.pone.0163335 (PMC5028034; doi:10.1371/journal.pone.0163335)

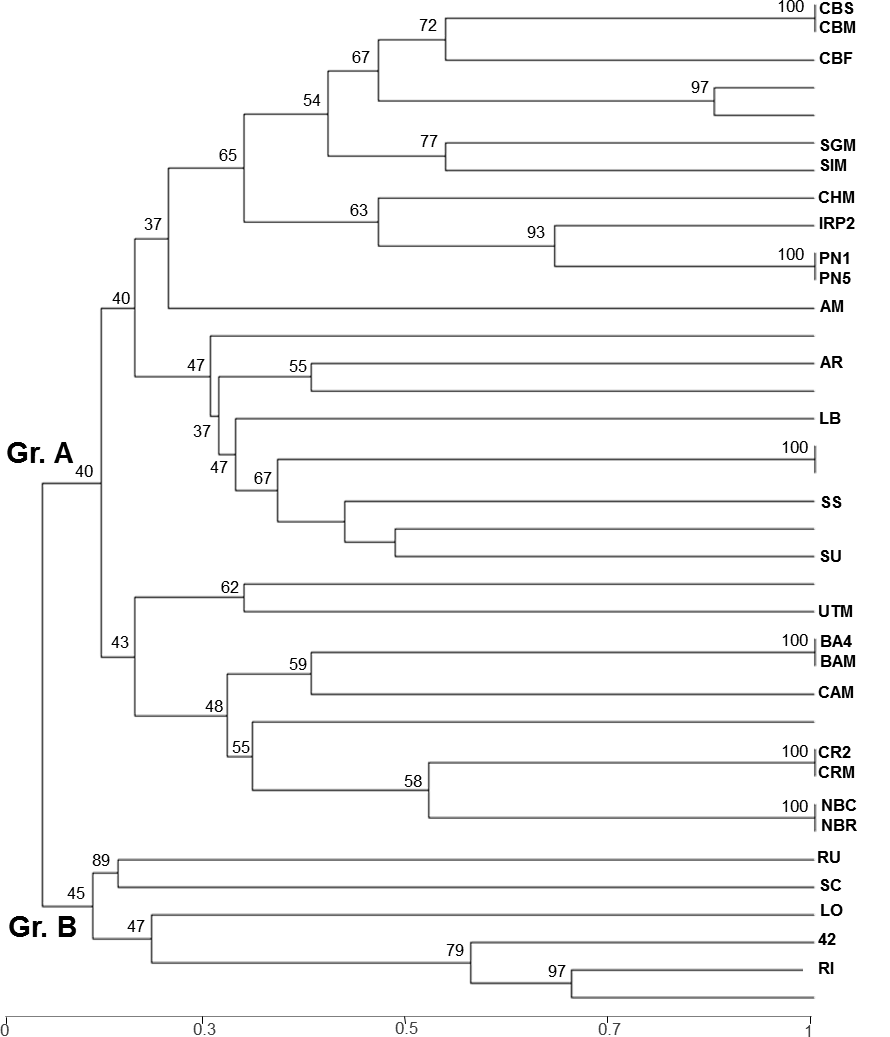

Supplement: S1 Fig — Dendrogram of the 37 grapevine accessions based on SSR marker analysis. The bar at the bottom of the figure provides a scale, from 0 to 1, of the estimated similarity. The numbers next to each node represent the bootstrap value (1000 replicates). (TIF) [file pone.0163335.s001.tif]

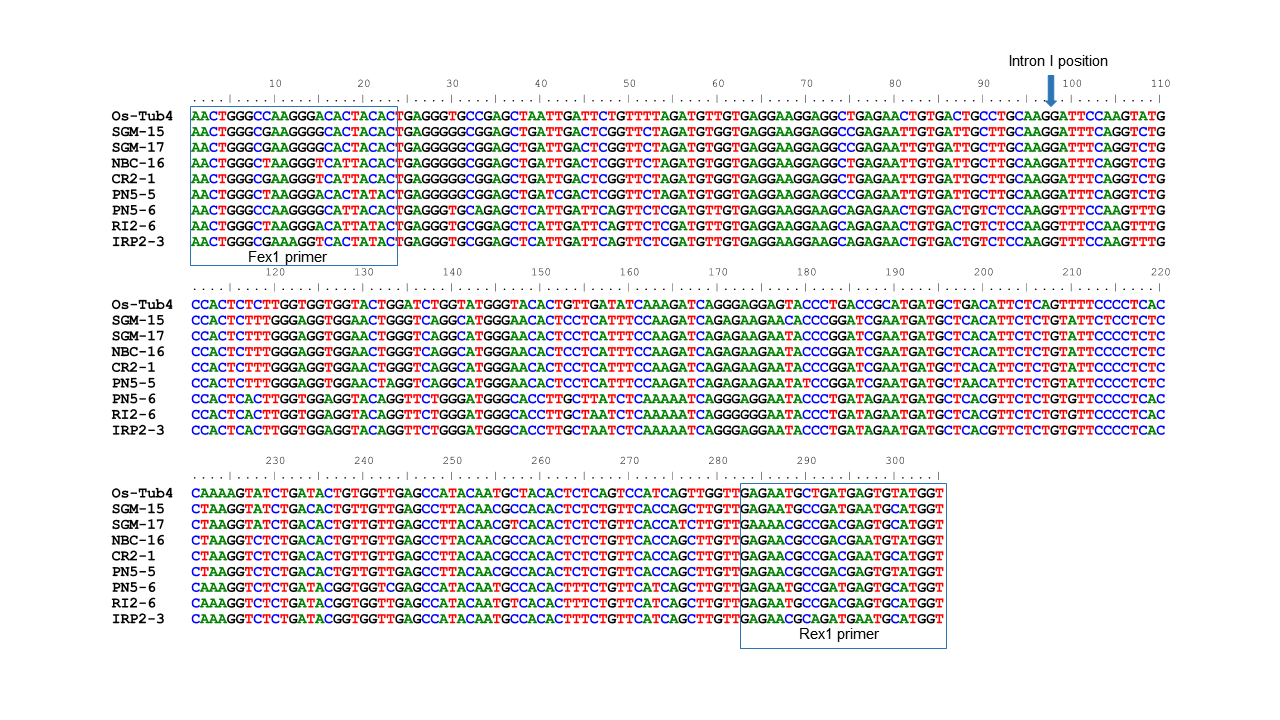

Supplement: S2 Fig — Exon nucleotide sequences amplified by TBP are aligned using rice OsTub4 as reference. The sequence corresponding to the primer pair (Fex1 and Rex1) is shown at the two opposite ends while an arrows indicate the position of intron1. (TIF) [file pone.0163335.s002.tif]

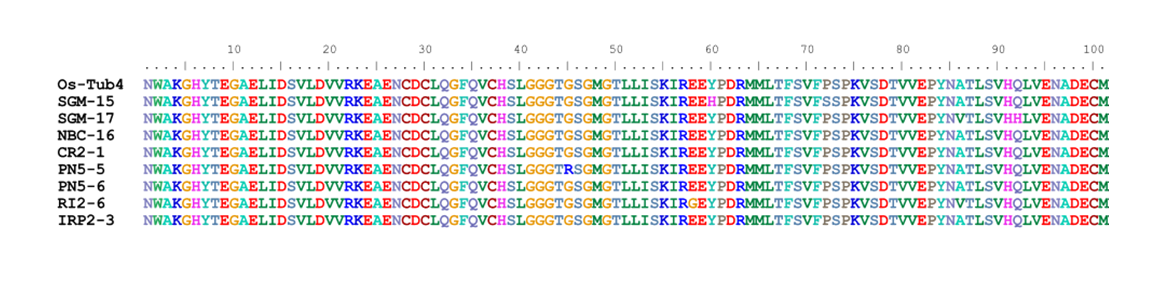

Supplement: S3 Fig — The tubulin exon portions shown in S2 Fig have been translated into the corresponding aminoacid sequences. (TIF) [file pone.0163335.s003.tif]
